# iTRAQ-Based Phosphoproteomic Analysis of *Toxoplasma gondii* Tachyzoites Provides Insight Into the Role of Phosphorylation for its Invasion and Egress

**DOI:** 10.3389/fcimb.2020.586466

**Published:** 2020-11-26

**Authors:** Cheng He, Mei-zhen Xu, Shuai Pan, Hui Wang, Hong-juan Peng, Zhuan-zhuan Liu

**Affiliations:** ^1^Jiangsu Key Laboratory of Immunity and Metabolism, Department of Pathogen Biology and Immunology, Xuzhou Medical University, Xuzhou, China; ^2^Department of Pathogen Biology, Guangdong Provincial Key Laboratory of Tropical Disease Research, School of Public Health, Southern Medical University, Guangzhou, China

**Keywords:** *Toxoplasma gondii*, iTRAQ, phosphoproteomic analysis, bioinformatic analysis, invasion, egress

## Abstract

The invasion and egress are two key steps in lytic cycle vital to the propagation of *Toxoplasma gondii* infection, and phosphorylation is believed to play important roles in these processes. However, the phosphoproteome of *T. gondii* at these two stages has not been characterized. In this study, we profiled the phosphoproteome of tachyzoites at the stages of “just invading” (JI) and “prior to egress” (PE) based on iTRAQ quantitative analysis, in which a total of 46 phosphopeptides, 42 phosphorylation sites, and 38 phosphoproteins were detected. In the comparison of PE vs. JI, 10 phosphoproteins were detected with their phosphorylation level significantly changed, and four of them were demonstrated to be significantly down-regulated at the transcriptional level. Bioinformatic analysis of these identified phosphoproteins suggested that phosphorylation-mediated modulation of protein function was employed to regulate the pathway of toxoplasmosis and metabolism and cellular processes correlated with tachyzoite’s binding, location, and metabolism, and thus play vital roles in the parasite lytic cycle. Moreover, cytoskeletal network (CN)-associated Inner Membrane Complex (IMC1, IMC4, IMC6 and IMC12), Intravascular Network (IVN)-related GRAs (GRA2, GRA3, GRA7 and GRA12), and Parasitophorous Vacuole Membrane (PVM)-localized ROP5 were shown to be enriched at the central nodes in the protein interaction network generated by bioinformatic analysis, in which the phosphorylation level of IMC4, GRA2, GRA3, and GRA12 were found to be significantly regulated. This study revealed the main cellular processes and key phosphoproteins crucial for the invasion and egress of *T. gondii*, which will provide new insights into the developmental biology of *T. gondii in vitro* and contribute to the understanding of pathogen-host interaction from the parasite perspective.

## Introduction

*Toxoplasma gondii* is an obligate intracellular apicomplexan parasite that chronically infects approximately one-third of the world’s human population, and the toxoplasmosis caused by its infection has been regarded as one of the major neglected parasitic infections (Hotez, 2014; Wei et al., 2016). Fortunately, most infections in healthy people do not show obvious clinical symptoms. However, severe complications, such as encephalitis and eye disease, even death can be caused by the infection of *T. gondii* in immunocompromised patients (Weiss and Dubey, 2009). Moreover, primary infection of *T. gondii* in pregnant women can be vertically transmitted to infect the fetus and result in miscarriage, premature birth, stillbirth, malformations, and other adverse pregnancy outcomes (Li et al., 2014). As an obligate intracellular parasite, the successful invasion and egress of *T. gondii* from its host cell are critical for survival, dissemination and transmission, and thus are believed to be essential for the propagation of parasite infection (Lavine and Arrizabalaga, 2007; Hortua Triana et al., 2018).

Phosphorylation is a key post-translational protein modification for regulating protein function, which is considered to regulate almost all aspects of cell life (Olsen et al., 2006; Schulze, 2010; Broncel and Treeck, 2020). The critical roles of phosphorylation in the lytic cycle of *T. gondii* have also been demonstrated in the previous studies. For example, preventing the phosphorylation of *Tg*IF2α with point mutation (S71A) lead to a significant delay in producing acute toxoplasmosis *in vivo* and a defect in adapting to the extracellular environment while the parasite searched for a new host cell *in vitro* (Joyce et al., 2010). Moreover, *Tg*MyoA is reported to regulate the initiation of motility and egress in the *T. gondii*’s lytic cycle, which largely depends on its phosphorylation (Gaji et al., 2015; Powell et al., 2018). Till now, however, only a few phosphorylated proteins of *T. gondii* have been identified, and the quantitative phosphoproteomic analysis of *T. gondii* at the different lytic cycle stages is also very few.

*T. gondii* invasion of its host is a rapid process, and to accomplish this process, complex signaling events within the parasite must occur. Meanwhile, the egress of *T. gondii* is an active process to rupture the PVM, which contributes to its dissemination and associates with the pathogenesis of its infection (Frénal et al., 2017). Here, the first 30 min post infection was defined as the phase of “just invasion”, when most of the *T. gondii* tachyzoites invade the host cell and the parasitophorous vacuoles (PVs) are newly formed. Moreover, since the tachyzoites have been thoroughly proliferated and are ready to egress at 28 h post infection (PI), this phase was termed as the phase of “prior to egress” (Treeck et al., 2011). In our study, we performed phosphoproteomic analysis of *T. gondii* to characterize the complicated events mediated by phosphorylation of *T. gondii* at these two infection stages, and also analyzed mRNA expression of the significantly regulated phosphoproteins that significantly changed between JI and PE. This current study will be helpful to elucidate the invasion and egress mechanisms of *T. gondii* and understand the role of phosphorylation in host-pathogen interactions from the perspective of the pathogen.

## Materials and Methods

### Cell and Parasite Culture

RH tachyzoites were maintained by serial passages in human foreskin fibroblast (HFFs) monolayers grown in Dulbecco’s modified Eagle’s medium (DMEM, Gibco) supplemented with 10% fetal calf serum (FBS; Gibco) and 100 μg/ml gentamicin.

### Sample Preparation

The HFF cells were infected with *T. gondii* RH tachyzoites with a multiplicity of infection (MOI) of 3 for 30 min and 28 h, respectively. After infection for 30 min, the unrecruited tachyzoites were washed off with phosphate buffered saline (PBS) for three times, and the JI groups were then harvested with cell scrapers. The PE groups were cultured for another 27.5 h and harvested with the same method. All six groups of cells were pelleted by centrifugation, stored in dry CO_2_, and sent to the Beijing Genomic Institute (BGI) for total protein extraction and subsequent analysis.

### Protein Extraction and Digestion

Protein extraction and digestion procedures were performed essentially as described previously (He et al., 2019). Briefly, the cells were suspended in lysis buffer (7 M urea, 2 M thiourea, 4% CHAPS, 40 mM Tris-HCl, pH 8.5, 1 mM PMSF, 2 mM EDTA) and sonicated on ice. The protein mixtures were precipitated by adding 4× volume of chilled acetone and leaving the mixtures overnight at -20°C. After centrifugation at 30,000 g and 4°C, each pellet was dissolved in 0.5 M triethylamine borane (TEAB; Applied Biosystems, Milan, Italy) and sonicated on ice. After centrifuging again at 30,000 g and 4°C, an aliquot of the supernatant was taken for determination of protein concentration by the Bradford method using BSA as a standard. For each example, 100 μg of the extracted proteins were digested at 37°C for 4 h with Trypsin Gold (Promega, Madison, WI, USA) with a protein-to-trypsin ratio of 40:1. Trypsin Gold was then added to each sample again with the same ratio, and the protein was digested for another 8 h. The digested peptides were desalted using a Strata X C18 column (Phenomenex, Torrance, CA, USA) and vacuum-dried.

### iTRAQ Labeling and Phosphopeptide Enrichment

The vacuum dried peptides from each group were reconstituted in 0.5 M TEAB and isotope labeling was carried out according to the manufacturer’s protocol for the iTRAQ Reagent 8-Plex Kit (Applied Biosystems, Foster City, CA, USA). The labeled samples were desalted using a Strata X C18 column (Phenomenex, Torrance, CA, USA) and vacuum-dried.

The dried peptides were reconstituted in a solution containing 65% (v/v) acetonitrile (ACN) and 3.5% (v/v) trifluoroacetic acid (TFA) and then saturated with glutamic acid. Phosphopeptides were enriched using TiO_2_, as previously described (He et al., 2017a). Briefly, iTRAQ-labeled peptides were added into the freshly prepared TiO_2_ beads (GL Sciences) at peptides-to-beads ratio of 1:4 (mass/mass) and then incubated for 20 min at 37°C with end-over-end rotation. The mixture was first washed with 65% ACN and 0.5% TFA (pH 2.0-3.5) and then with 65% ACN and 0.1% TFA (pH 2.0–3.5). The phosphopeptides were eluted with 0.3 M NH_4_OH solution in 50% (v/v) ACN (diluted from a 25% NH_4_OH solution) and vacuum-dried.

### Peptide Fractionation

The enriched peptide mixtures were reconstituted with 300 μl 1%TFA and separated according to the manufacturer’s protocol for the High pH Reversed-Phase Peptide Fractionation Kit (Thermo Scientific Pierce, #84868). The eluted peptides were pooled into six fractions and vacuum-dried.

### Liquid Chromatography-Tandem Mass Spectrometry Analysis Using Q Exactive

Each fraction was resuspended in buffer A (2% ACN, 0.1% FA) and centrifuged at 20, 000 g for 10 min. The supernatant was loaded by the auto sampler onto a trap column on a LC-20AD nanoHPLC (Shimadzu, Kyoto, Japan) for trapping and desalting. The peptides were then eluted onto a 15 cm analytical C18 column (inner diameter 75 μm, column particle size 3.6 μm) that was packed in-house. The samples were loaded and subjected to the following conditions: 8 min at a flow rate of 300 nl/min maintaining at 5% buffer B (98% ACN, 0.1% FA), followed by 68 min linear gradient to 21%, 6 min linear gradient to 32%, and 3 min linear gradient to 80%, and then maintenance at 80% buffer B for 5 min, and finally a return to 5% buffer B for 5 min.

The peptides were then subjected to ionization with nano-electrospray ionization (nanoESI) followed by analysis with a Q Exactive Tandem Mass Spectrometer (Thermo Fisher Scientific, MA, USA) in a data-dependent acquisition mode. The electrospray voltage applied was 1.6 kV. MS1 spectra were collected in the range 350–1,500 m/z at a resolution of 70,000 and MS2 spectra were collected in the fixed starting 100 m/z at a resolution of 17,500. The 20 most intense precursors with a charge state of 2+ to 5+ were selected for MS2 fragmentation with 20 s dynamic exclusion setting. Peptides were selected for MS2 using the high-energy collision dissociation operating mode with a normalized collision energy setting of 30, and the ion fragments were detected in the Orbitrap. The AGC target value for MS1 and MS2 was set at 3E6 and 1E5, respectively.

### Phosphoproteomic Data Analysis

For iTRAQ phosphorylated protein identification, raw MS/MS spectra were processed with Proteome Discoverer 1.4 (Thermo Fisher Scientific) and searched using in-house Mascot 2.3 (Matrix Science, London, UK) against the download protein sequences from *Toxo*DB database (https://toxodb.org/toxo/). The search parameters were as follows: enzyme, trypsin; peptide mass tolerance, 20 ppm; fragment mass tolerance, 0.05 Da; fixed modifications, Carbamidomethyl (C), iTRAQ8plex (N-term), iTRAQ8plex (K); variable modifications, Oxidation (M), Acetyl (Protein N-term), Deamidated (NQ), Phospho (ST), Phospho (Y), and iTRAQ8plex (Y); and max missed cleavage, 2. The search results were further processed using Percolator and a peptide false discovery rate (FDR) ≤0.05 was used as the criterion for defining confidential peptides. The phosphorylation sites of identified phosphopeptides were scored with Proteome Discoverer applying in-house phosphoRS 3.1, and the confidence was set at a phosphoRS site probability ≥0.75 (Taus et al., 2011). For iTRAQ quantification, the peptide for quantification was automatically selected by the algorithm to calculate the reporter peak area, error factor (EF), and p-value (default parameters in Mascot software package). Student’s t-test was performed using the Mascot software. The resulting data set was auto bias-corrected to the biological replicates. The peptide ratios were normalized by dividing by the median ratio of all the peptides identified (Wu et al., 2015). A phosphoprotein with a between-group 1.5-fold change in phosphorylation level at p ≤0.05 was considered to be a significantly regulated phosphoprotein (Nguyen et al., 2012).

### Functional Analysis of the Phosphorylation Data Set

All the identified phosphoproteins between the two comparison groups (JI and PE) were subjected to bioinformatic analysis. Gene Ontology (GO) annotation and enrichment analysis were completed using the web-based GO software (http://www.geneontology.org) (Ashburner et al., 2000). A pathway analysis was performed at the Kyoto Encyclopedia of Genes and Genomes database (KEGG, http://www.genome.p/kegg) (Kanehisa and Goto, 2000). Hypergeometric tests were used for identifying significantly enriched GO terms and KEGG pathways. A significance level of p <0.05 was used as the enrichment cut off threshold for GO terms and KEGG pathways (Nguyen et al., 2012). All the identified proteins were used as the backgrounds of GO and KEGG analyses with all the identified *T. gondii* phosphorylated proteins. Additionally, interaction networks for all the identified phosphoproteins in these two groups were conducted using STRING 11.0 (http://string-db.org).

### Transcript Expression Analysis With qPCR

Total RNA was isolated from HFF cells infected with *T. gondii* for 30 min and 28 h by Trizol (Invitrogen), and then reversely transcribed using Reverse Transcriptase (*Vazyme*). Each biological replicate was analyzed in triplicates by SYBR green-based quantitative real-time PCR using Top Green qPCR SuperMix (*TransGen* Biotech) on Light Cycler 480II (*Roche*) with the primers shown in **Table S1**. Mean fold-changes from three independent experiments were calculated from *ΔΔCT* values using actin transcript as a housekeeping gene (Buguliskis et al., 2010; Blume et al., 2015).

## Results

### Phosphoproteomic Identification of *T. gondii* Tachyzoites at Different Infection Phases

**Figure 1:**
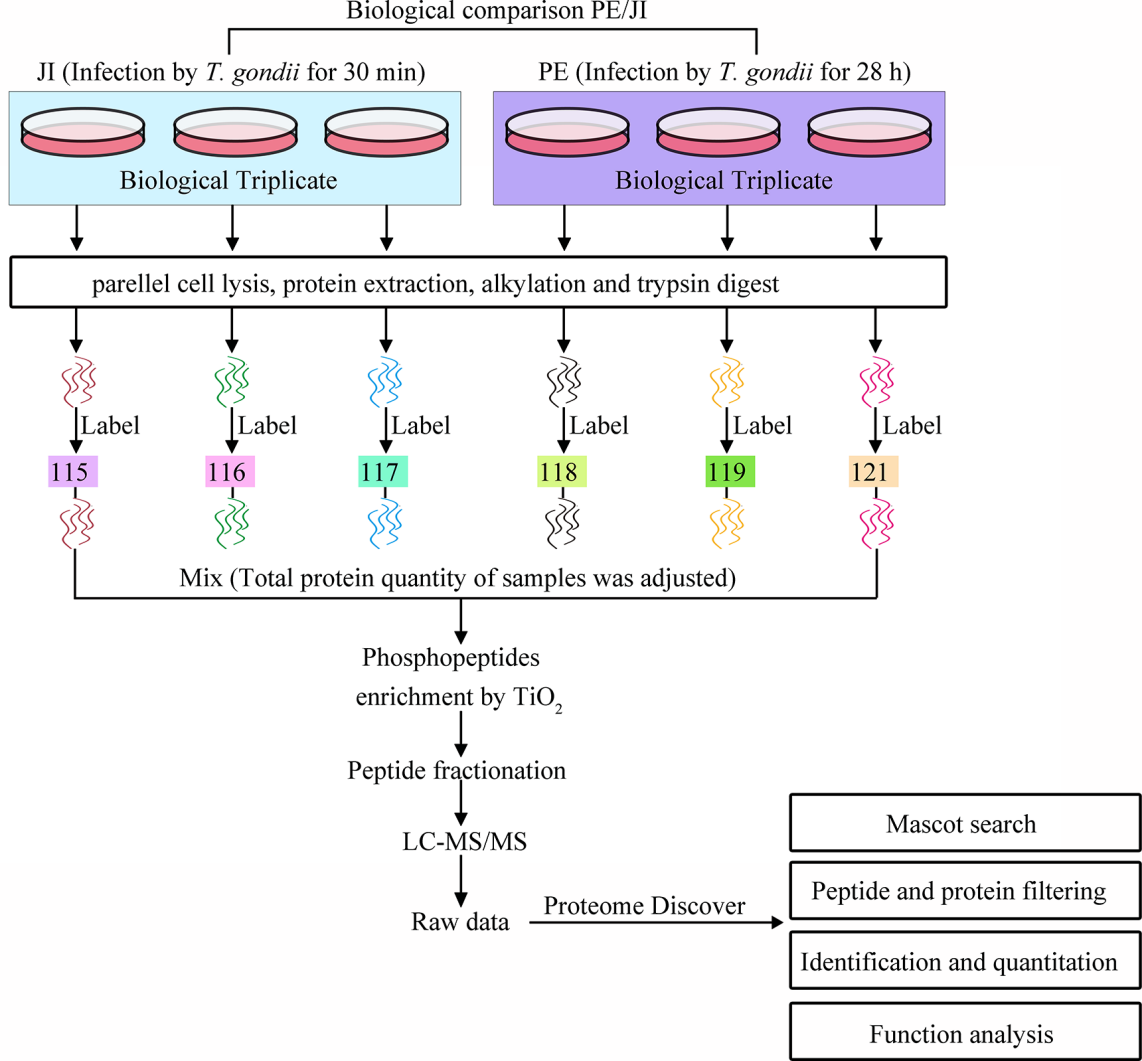
Processes of the iTRAQ-based phosphoproteomic analysis of *T. gondii* tachyzoites at the stages of “just invading” and “prior to egress”. Two biological triplicates of cells infected with *T. gondii* RH tachyzoites for 30 min or 28 h, respectively, at a MOI of 3 were collected for the phosphoproteomic analysis of *T. gondii* tachyzoites. All the six samples were digested with trypsin and labeled with iTRAQ. The labeled phosphopeptides were enriched with TiO2, and subjected to LC-MS/MS analysis. The generated phosphoproteomic data were then qualitatively, quantitatively, and functionally analyzed.

**Figure 2:**
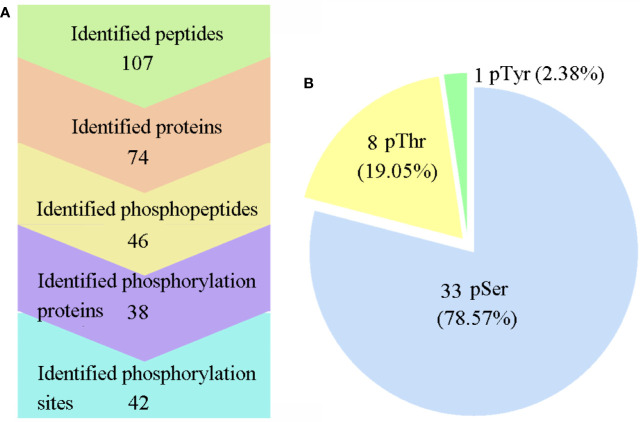
Large-scale mass spectra information regarding the phosphoproteome data. **(A)** Information on the identified proteins, phosphoproteins, peptides, phosphopeptides, and phosphosites. **(B)** Distribution of the pSer/pThr/pTyr phosphoproteome. Phospho-Ser was the most abundant site, and it accounted for 78.57% of all phosphorylated amino acids, followed by phospho-Thr (19.05%) and phospho-Tyr (2.38%).

All six samples were labeled with iTRAQ reagents, and the phosphopeptides were enriched by TiO_2_, which were then analyzed with LC-MS/MS. The workflow of this study is presented in **Figure 1**. As a result, a total of 46 phosphopeptides matching to 38 phosphoproteins were identified with a false-discovery rate (FDR) ≤0.01 in phosphopeptide level, and 42 phosphorylation sites were detected with phosphoRS probability ≥0.75 in phosphorylation site level (**Figure 2A**). The detected 42 phosphorylation sites consisted of 33 (78.57%) serine phosphorylation (pSer), 8 (19.05%) threonine phosphorylation (pThr), and 1 (2.38%) tyrosine phosphorylation (pTyr) (**Figure 2B**). The detailed information of all the identified phosphoproteins is shown in **Table S2**.

### Identification and Quantification of the Significantly Regulated Tachyzoite Phosphoproteins

The phosphopeptides identified in the two groups (JI and PE) were quantified using Mascot software, and between these two groups, the comparative analysis of phosphorylation level change was further performed based on PE/JI ratio. The comparative phosphorylation level analysis led to the identification of 8 up-regulated (TGGT1_311480, GRA12, IMC4, TGGT1_273460, TGGT1_230940, GRA2, TGGT1_257530, GRA3) and 2 down-regulated phosphoproteins (TGGT1_239800, TGGT1_228360) (p < 0.05). The detailed information of phosphoproteins with phosphorylation level significantly changed is shown in **Table S2**.

### Function Analysis of the Identified Phosphoproteins

**Figure 3:**
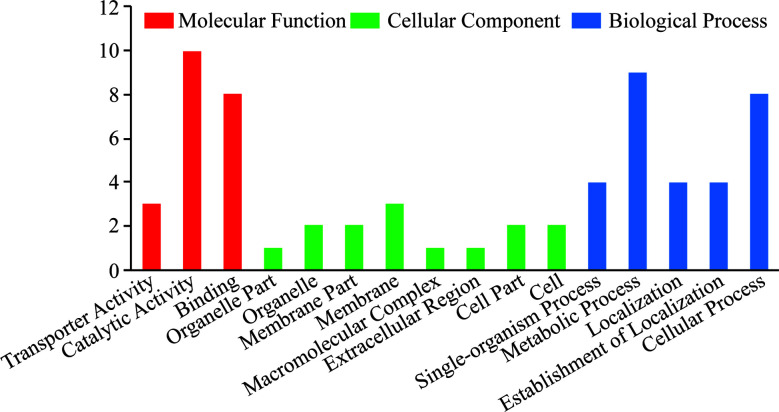
GO enrichment analysis of all the identified *T. gondii* phosphoproteins. GO analysis of all the 38 identified phosphoproteins revealed that proteins in the “biological process”, “cellular component”, and “molecular function” categories were enriched. These phosphoproteins were suggested to be involved in the regulation of cellular processes including binding, location, and metabolism correlated with *T. gondii* invasion and egress.

To gain a better understanding of the phosphoproteins’ role in the lytic cycle processes of *T. gondii* tachyzoites, we performed GO and KEGG enrichment analysis with the identified phosphoproteins. The enriched GO terms were assigned to molecular function (MF), biological process (BP), cellular component (CC), and the results are shown in **Figure 3**. The terms of the MF were significantly enriched with the identified phosphoproteins including “transporter activity”, “catalytic activity”, and “binding”. In the BP, the terms were significantly enriched with the identified phosphoproteins including “single-organism process”, “metabolic process”, “localization”, “establishment of localization”, and “cellular process”. While in the CC, terms of “organelle part”, “organelle”, “membrane part”, “membrane”, “macromolecular complex”, “extracellular region”, “cell part”, and “cell” were significantly enriched. Unfortunately, since the number of identified phosphoproteins with phosphorylation level significantly changed was few, no term in MF and CC was significantly enriched, except for the terms of “single-organism cellular process”, “single-organism process”, and “cellular process” in BP enriched with the significantly regulated phosphoproteins.

**Figure 4:**
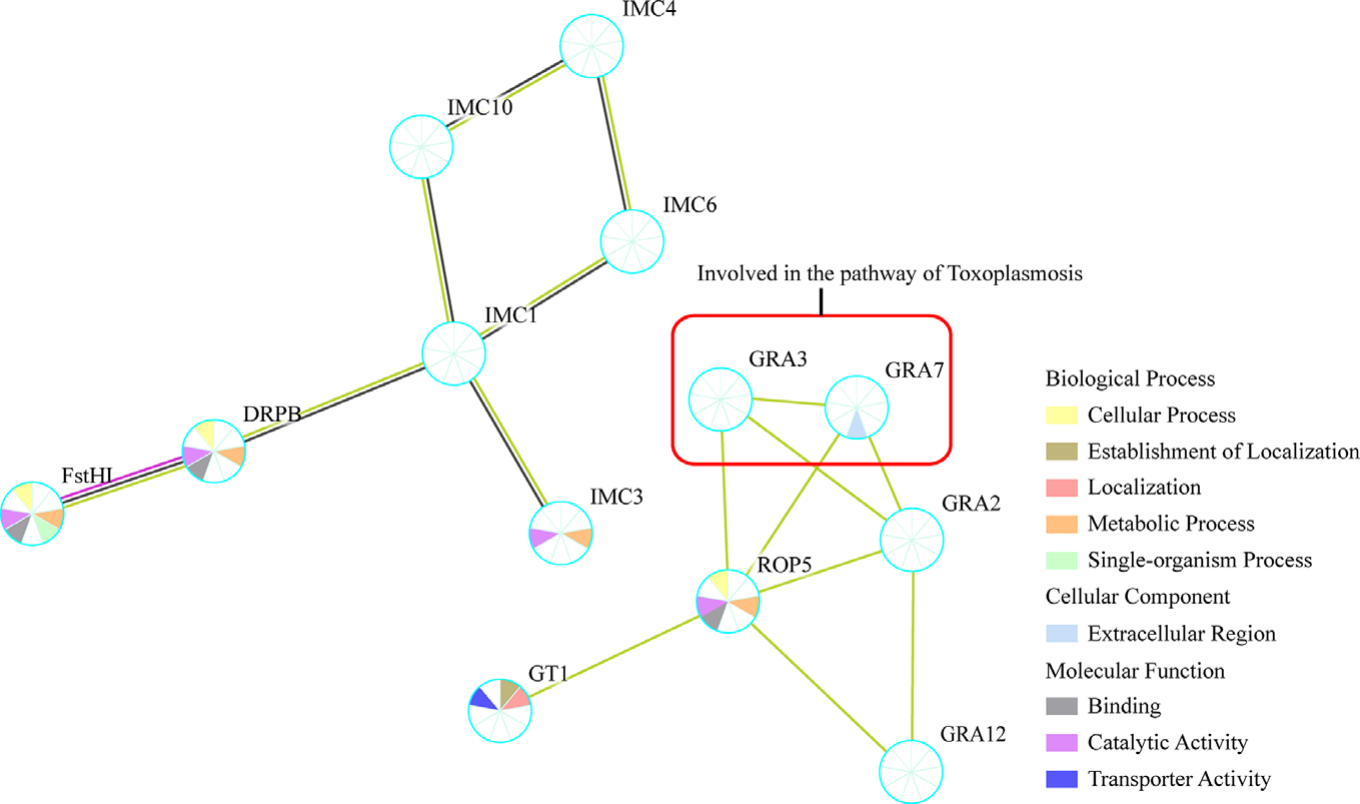
Protein–protein interaction (PPI) networks of all the identified phosphoproteins. The PPI network was generated with the identified tachyzoites phosphoproteins *via* STRING v11.0 with a confidence score over 0.7. GRA3 and GRA7 within red box were suggested to be involved in the regulation of Toxoplasmosis pathway.

The KEGG pathway analysis with all the identified phosphoproteins revealed that the enriched pathways were closely associated with the pathogenicity of *T. gondii*, such as “toxoplasmosis”, “metabolic pathways”, and “glycerophospholipid metabolism” (**Table S3**, **Figure S1**). For the same reason with GO analysis, however, only one pathway correlated with “toxoplasmosis” was enriched with the significantly regulated phosphoproteins in the comparison group of PE vs. JI (**Figure 4**).

### Protein–Protein Interaction Analysis

The protein–protein interaction (PPI) network of the identified phosphoproteins were analyzed using STRING 11.0, and the resulting PPI network included 16 nodes and 37 edges and was then constructed by removing unconnected proteins and self-loops as shown in **Figure 4**. The PPI network was constructed by setting the minimum required interaction score to medium confidence (0.4). Based on the results of PPI network analysis, phosphoproteins of GRA2, GRA3, GRA7, GRA12, and ROP5 were shown to be enriched at the central nodes in the PPI network, which were demonstrated to be important to suppress the immune recognition by the host cell (Rommereim et al., 2019). In addition, some other phosphoproteins with unknown function such as IMC1, IMC4, IMC6, IMC12, and GT1 were also shown as important node proteins in the PPI network (**Figure 4**).

### Transcription Level Assay of the Significantly Regulated Phosphoproteins

**Figure 5:**
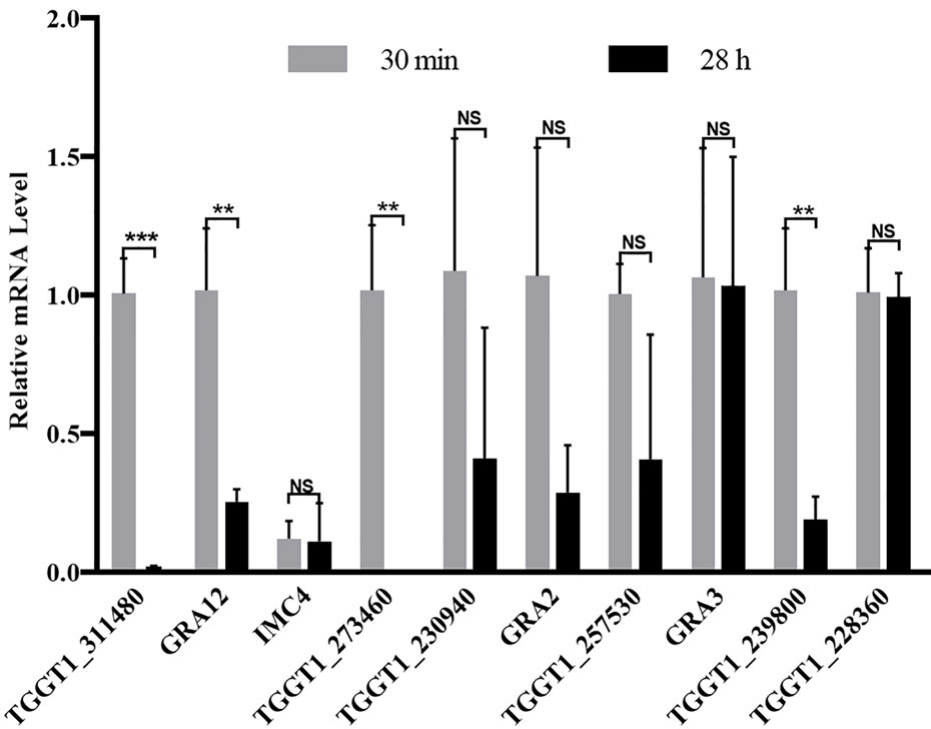
Relative transcription level of the genes with their protein’s phosphorylation level significantly changed. The transcription level of ten phosphoproteins with the phosphorylation level significantly changed were detected with qPCR, ant the Actin was used as an internal control. Error bars represent SD from three independent replicates, and statistical values were determined by t test. **p < 0.01; ***p < 0.001; NS, no significant difference.

The transcription level of ten phosphoproteins with phosphorylation level significantly changed in the comparison group of PE vs. JI were analyzed with qPCR. Four of the ten *T. gondii* phosphoproteins (TGGT1_311480, GRA12, TGGT1_273460, and TGGT1_239800) were demonstrated to be significantly down-regulated at the transcription level, while the other 6 phosphoproteins were revealed with no significant change (**Figure 5**).

## Discussion

The invasion and egress of *T. gondii* tachyzoites are two basic processes of the lytic cycle, which entails complex signaling events within both the parasite and its host (Treeck et al., 2011; Blader et al., 2015). As a key post-translational modification in mediating the protein function, the phosphorylation was considered to be involved in regulating almost all the signaling events, and the phosphorylation has been proved to play important roles in regulating the invasion and egress processes of *T. gondii* (Tang et al., 2014; Krishnamurthy et al., 2016; Stewart et al., 2016; Wallbank et al., 2019). For example, phosphorylation of host Irga6 by *Tg*ROP18 inhibits its accumulation and action on the PVM, thereby promoting *T. gondii* escape from host immune clearance (Steinfeldt et al., 2010). Moreover, the phosphorylation-mediated activation of host STAT3 induced by *Tg*ROP16 enhances the production of proinflammatory cytokines, including IL-6 and IL-12, to eliminate the parasites (Yamamoto et al., 2009). Corresponding to the essential roles of phosphorylation in modifying the function of a host protein, the crucial role of phosphorylation of *T. gondii* protein in mediating the invasion and egress processes of *T. gondii* has also been reported (Tang et al., 2014; Krishnamurthy et al., 2016). For example, Tang’s group has demonstrated that enhanced phosphorylation of *Tg*MyoA increases the efficiency of invasion and accelerates the process of calcium-induced egress (Tang et al., 2014). Additionally, the dephosphorylation of *Tg*AMA1 is demonstrated to be necessary for the optimal invasion of *T. gondii* (Krishnamurthy et al., 2016). Therefore, phosphorylation is a double-edged sword, which can play both positive and negative regulatory roles in mediating the interaction between *T. gondii* and its host. Recently, iTRAQ based phosphoproteomic quantitative analysis has emerged as an efficient approach to screen key phosphoproteins essential for the pathogenesis of *T. gondii*. Intriguingly, our previous iTRAQ-based phosphoproteomic analysis with respect to the host cell at the infection stages of JI and PE manifests that *T. gondii* manipulates host cell processes including apoptosis, metabolism, cytoskeleton reorganization through phosphorylation (He et al., 2019). Based on the results of our phosphoproteomic analysis, we found that the phosphorylation levels of host cell vimentin were significantly modulated by *T. gondii* at the two infection stages. Further functional analysis revealed that vimentin inhibits the invasion of *T. gondii* (He et al., 2017b). Consequently, the phosphoproteomic analysis of *T. gondii* at the infection phases of JI and PE may provide new clues to unravel the mechanisms controlling the parasite’s invasion and egress processes. However, the phosphoproteome data and its quantitative information with respect to *T. gondii* tachyzoite at the infection phases of just invasion and prior to egress have not been identified yet.

With the illustration of the vital roles of phosphorylation for *T. gondii* infection, the phosphoproteomic analysis of *T. gondii* has been gradually explored. For example, Wang et al. analyzed the phosphoproteome of three different strains *of T. gondii* tachyzoites (RH strain, PRU stain, and PYS strain), and identified several hundreds of phosphoproteins. This phosphoproteomic analysis is therefore beneficial for explaining the contribution of phosphorylation in regulating the virulence heterogeneity of the three parasite strains (Wang et al., 2019). In our study, however, only 38 phosphoproteins were identified, which were much less than the reported number of phosphoproteins in *T. gondii* tachyzoites. The discrepancy between the number of identified *T. gondii* phosphoproteins may be due to the far below amount of loading protein of *T. gondii* for the analysis performed with the same approach, completely different lytic cycles of tachyzoites and various sources of parasite collection (Wang et al., 2019). Since the phosphoproteome data generated in this study was confirmed by western blot from the aspect of human cells (He et al., 2019), the phosphoproteome data reported in this study therefore is very reliable. Interestingly, most of these identified phosphoproteins were revealed with low mean phenotype scores based on the genome-wide CRIPSR screened results performed by Sidik’s group (**Table S2**), which suggested that the phosphorylation could be employed to modulate these protein’s function thereby controlling the invasion and egress processes of *T. gondii* (Sidik et al., 2016). Additionally, among the ten significantly regulated phosphoproteins, four of them were demonstrated to be significantly down-regulated at the transcriptional level in the comparison group of PE vs. JI, which suggested that some phosphoproteins were involved in the regulation of the invasion and egress processes of *T. gondii* not only through modifying the phosphorylation status, but also by regulating the mRNA expression.

The results of the bioinformatic analysis in our study showed that these identified phosphoproteins were mainly involved in the regulation of the processes of metabolism and location, and their function emphasized on “transporter activity”, “catalytic activity”, and “binding”. Our analysis is in agreement with previous studies, which demonstrated that multiple phosphoproteins played crucial roles in modulating the enriched biological processes. For example, GRA2 is reported to be involved in the formation of IVN in the PV, which allows nutrients transportation to nourish the parasites (Nam, 2009). Deletion of GRA2 lead to failed formation of the IVN and also decreases the infection rate *in vitro* (Rommereim et al., 2016). Intriguingly, the phosphorylation level of GRA2 was detected to be up-regulated significantly in the comparison group of PE vs. JI, which suggested that phosphorylation may be one of the important mechanisms in mediating the function of GRA2. Surprisingly, both GRA3 and GRA12 were identified with enhanced phosphorylation level in this study yet were shown to be dispensable for the lytic cycle of *T. gondii in vitro*, however, they play essential roles in acute infection of *T. gondii in vivo* (Craver and Knoll, 2007; Rommereim et al., 2016; Fox et al., 2019; Wang et al., 2020). The explanation of this discrepancy may be that GRA3 and GRA12 do not play their roles independently in the lytic cycle process of tachyzoites but interdependently, just as other GRA proteins (Rommereim et al., 2016). Consequently, double knockout stains of GRA3 and GRA12, or even more GRAs, need to be generated to explore their roles in the lytic cycle of *T. gondii*. Moreover, another significantly regulated phosphoprotein, ApiAT5-3 (TGGT1_257530), identified in this work has been shown to play an essential role in the lytic cycle of *T. gondii* other than egress, despite it being rapidly phosphorylated at Ser56 after induction of egress with ionophore (Wallbank et al., 2019). Wallbank et al. reports that the phosphorylation of ApiAT5-3 at the process of PE is prepared for the extracellular milieu, or reinvasion of tachyzoites. However, how the phosphorylation of ApiAT5-3 functions during the lytic cycle of *T. gondii*, and whether there are other phosphorylation sites involving in the regulation of its function need to be further explored. In our study, we found the phosphorylation level of ApiAT5-3 at Ser24 was significantly up-regulated prior to egress compared to the stage of just invasion of *T. gondii*. This may provide new insight into the mechanism of how ApiAT5-3 works in the lytic cycle of *T. gondii*.

KEGG pathway analysis showed that the identified phosphoproteins were annotated into pathways with direct relevance to toxoplasmosis and metabolism, such as “Glycerophospholipid metabolism” and “Metabolic pathways” (**Table S3**, **Figure S1**), which is consistent with the reported phosphoproteins function in the phosphoproteomic analysis of different *T. gondii* strains (Wang et al., 2019). This result suggests that phosphoprotein-mediated pathway regarding toxoplasmosis and metabolism may be critical for the pathogenesis of *T. gondii*. Further analysis of the key phosphoproteins involved in the regulation of these shared pathways may provide potential clues to illustrate the mechanism of the invasion and egress, such as GRA3, GRA7, and TGGT1_306540. Moreover, five phosphoproteins ROP5, GRA7, GRA2, GRA3, and GRA12, were shown to locate at the central nodes in the PPI network. All these five phosphoproteins were demonstrated to play essential roles in reducing T cell recognition (Rommereim et al., 2019), which could further avoid the clearance of parasite by the host immune system. What is interesting is that three of the five phosphoproteins, GRA2, GRA3, and GRA12, were found to be significantly up-regulated at the phosphorylation level in the comparison group of PE vs. JI. Some of other phosphoproteins were shown to locate at the central nodes in the PPI network, such as IMC1, IMC4, and IMC12, and they may have important roles in the regulation of *T. gondii* lytic cycle. Unfortunately, the biological significance of these identified phosphorylation has not been explored yet. Therefore, further work is required to unravel the function of these identified phosphoproteins in the process of parasite lytic cycle.

In conclusion, iTRAQ-based phosphoproteomic analysis in this research will supplement the phosphoproteome data of *T. gondii* at the different lytic cycle stages. Moreover, the identified phosphoproteins and enriched cellular processes will contribute to understanding the mechanism of *T. gondii* invasion and egress and future studies should aim to clarify the precise function of these identified phosphoproteins as well as characterize the phosphoproteome data of other lytic cycle processes besides the present two stages.

## Data Availability Statement

All datasets generated for this study are included in the article/**Supplementary Material**. All the original mass spectrometry data have been deposited to the ProteomeXchange Consortium (http://proteomecentral.proteomexchange.org) *via* the iProX partner repository (Ma et al., 2019) with the dataset identifier PXD020655.

## Author Contributions

CH performed the experiments, analyzed the data, and wrote the paper. MX and SP performed the experiments and data analysis. HW revised the paper. HP and ZL helped conceive and design the experiments. ZL provided advice on data interpretation and edited the paper. All authors contributed to the article and approved the submitted version.

## Funding

This research was provided by the Natural Science Foundation of Jiangsu Province (BK20190983), the Natural Science Foundation of the Jiangsu Higher Education Institutions of China (19KJB310022), and the Research Foundation of Xuzhou Medical University (D2019018) to CH.

## Conflict of Interest

The authors declare that the research was conducted in the absence of any commercial or financial relationships that could be construed as a potential conflict of interest.

## Supplementary Material

Supplementary Figure 1 Enriched KEGG pathways with all the identified phosphoproteins in the comparison group of PE vs. JI. Yellow nodes represent the identified phosphoproteins involved in the regulation of enriched KEGG pathways, which was denoted with the surrounding blue nodes. Protein with red color indicated the phosphoprotein with the phosphorylation level significantly changed.

Supplementary Table 1 qPCR primers for the transcription level detection of the genes with their protein’s phosphorylation level significantly changed.

Supplementary Table 2 Quantitative and the mean phenotype score information of all the identified *T. gondii* phosphoproteins.

Supplementary Table 3 Enriched KEGG pathways with all the identified *T. gondii* phosphoproteins and the information of phosphoproteins involved in the regulation of these pathways.
