# Supplementary figures and images for: iTRAQ-Based Phosphoproteomic Analysis of Toxoplasma gondii Tachyzoites Provides Insight Into the Role of Phosphorylation for its Invasion and Egress
Source: Front Cell Infect Microbiol. 2020 Nov 26;10:586466. doi: 10.3389/fcimb.2020.586466 (PMC7756149; doi:10.3389/fcimb.2020.586466)

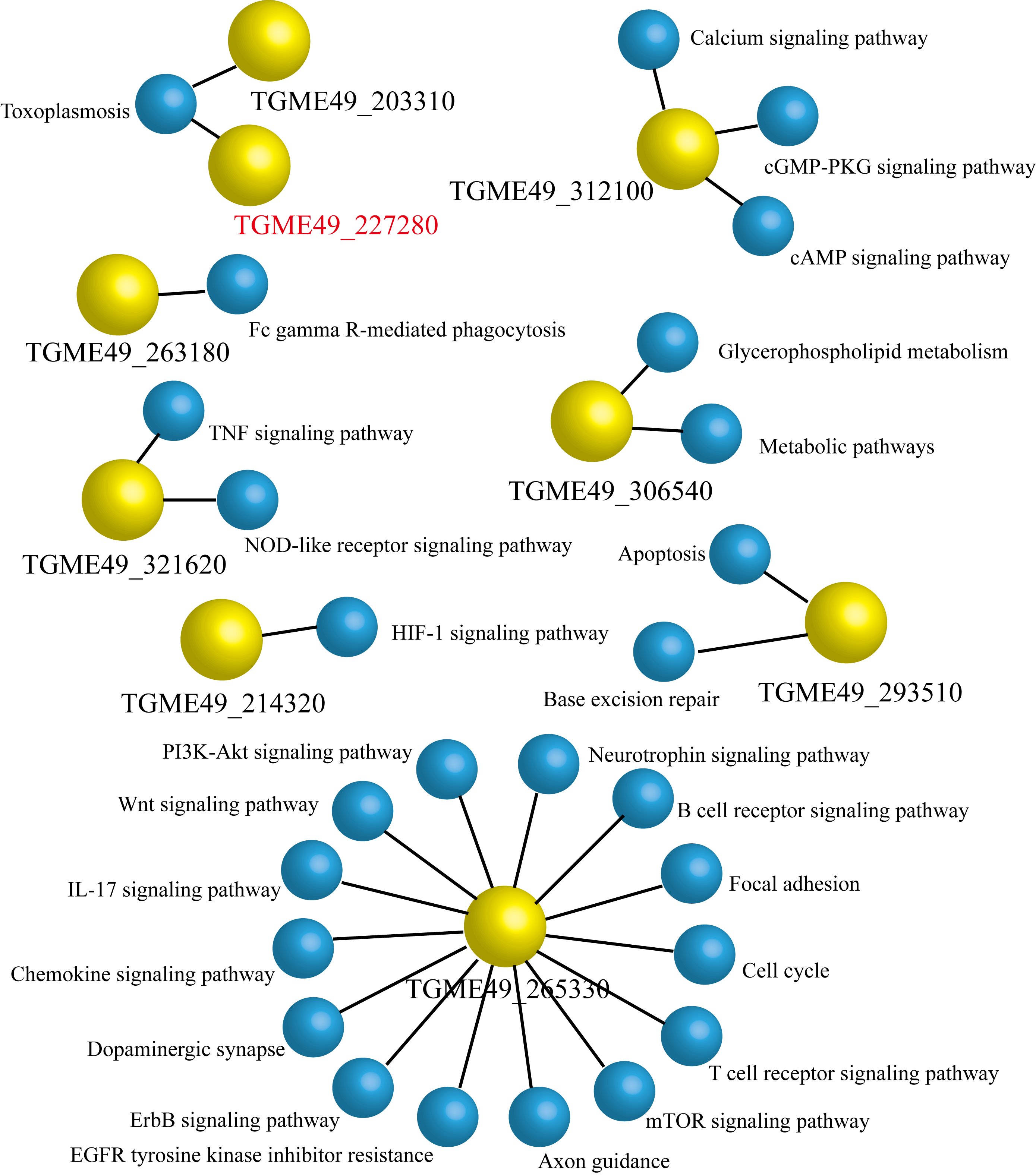

Supplement: Supplementary Figure 1 — Enriched KEGG pathways with all the identified phosphoproteins in the comparison group of PE vs. JI. Yellow nodes represent the identified phosphoproteins involved in the regulation of enriched KEGG pathways, which was denoted with the surrounding blue nodes. Protein with red color indicated the phosphoprotein with the phosphorylation level significantly changed. [file Image_1.tif]
